# Atypical Imaging Findings in a Cortisol-producing Adrenal Adenoma Predominantly Composed of Lipid-poor Compact Cells

**DOI:** 10.1210/jcemcr/luae189

**Published:** 2024-10-18

**Authors:** Satoshi Kawata, Yoshinari Obata, Aya Akai-Samoto, Kosuke Mukai, Kazuyuki Miyashita, Iichiro Shimomura

**Affiliations:** Department of Metabolic Medicine, Graduate School of Medicine, Osaka University, Suita, Osaka 565-0871, Japan; Department of Metabolic Medicine, Graduate School of Medicine, Osaka University, Suita, Osaka 565-0871, Japan; Department of Metabolic Medicine, Graduate School of Medicine, Osaka University, Suita, Osaka 565-0871, Japan; Department of Metabolic Medicine, Graduate School of Medicine, Osaka University, Suita, Osaka 565-0871, Japan; Department of Metabolic Medicine, Graduate School of Medicine, Osaka University, Suita, Osaka 565-0871, Japan; Department of Metabolic Medicine, Graduate School of Medicine, Osaka University, Suita, Osaka 565-0871, Japan

**Keywords:** Cushing syndrome, compact cell, CT, scintigraphy, PET-CT

## Abstract

Cortisol-producing adrenal adenomas consist of a mixture of lipid-rich clear and lipid-poor compact cells in varying proportions. Most adenomas are mainly composed of lipid-rich clear cells and typically exhibit low computed tomography (CT) attenuation values, high uptake on ^131^I-adosterol scintigraphy, and mild accumulation on ^18^F-fluorodeoxyglucose positron emission tomography/CT. However, adenomas predominantly composed of lipid-poor compact cells are rare, with limited evidence regarding their imaging characteristics. A 27-year-old woman with weight gain and a moon face was referred to our hospital. She presented with hypertension, dyslipidemia, low plasma ACTH levels, and autonomous cortisol secretion. We diagnosed this patient with ACTH-independent Cushing syndrome with a left adrenal tumor. The adrenal tumor exhibited imaging findings atypical for an adenoma, including high CT attenuation values, negative uptake on ^131^I-adosterol scintigraphy, and strong accumulation on ^18^F-fluorodeoxyglucose positron emission tomography/CT. Histopathological analysis indicated that the tumor was a rare type of adenoma, predominantly composed of lipid-poor compact cells. These findings and recent reports suggest that the proportion of compact cells can influence the imaging findings. Therefore, in the differential diagnosis, it is important to recognize that cortisol-producing adrenal adenomas predominantly composed of compact cells can present with atypical imaging findings.

## Introduction

ACTH-independent Cushing syndrome arises from excessive cortisol production by adrenal tumors, primarily benign adenomas [1]. Cortisol-producing adrenal adenomas consist of a mixture of lipid-rich clear and lipid-poor compact cells in varying proportions, with the lipid content depending on the ratio of these 2 cell types. Most adenomas are mainly composed of clear cells with abundant intracellular lipids, resulting in low unenhanced computed tomography (CT) attenuation values (typically <10 Hounsfield units) because of their high lipid content [2, 3]. ^131^I-adosterol scintigraphy is commonly used in Japan, showing high uptake in cortisol-producing adenomas [4]. Adrenocortical carcinomas can also overproduce cortisol, displaying high unenhanced CT attenuation values, and sometimes negative uptake on ^131^I-adosterol scintigraphy [5, 6]. To distinguish between benign adenomas and carcinomas, ^18^F-fluorodeoxyglucose positron emission tomography/CT (^18^F-FDG PET/CT) is reported to be a useful tool [7]. Unlike typical clear cell-rich adenomas, 20% to 30% of adenomas have a higher proportion of compact cells with poor lipid content, resulting in a lipid-poor appearance. However, adenomas predominantly composed of compact cells are rare [3], and there is limited evidence regarding their imaging characteristics, posing challenges for diagnosis. Here, we present a case of a young woman with ACTH-independent Cushing syndrome resulting from a compact cell-dominant adenoma who exhibited atypical imaging findings. The relationship between the postoperative histopathological analyses and imaging results is also discussed.

## Case Presentation

A 27-year-old woman with a left adrenal tumor was admitted to our hospital. She had no medical history until the age of 24 years, when she was diagnosed with hypertension and dyslipidemia during a medical checkup and experienced weight gain. At age 25 years, she began treatment with azilsartan (an angiotensin II receptor blocker) and esaxerenone (a selective mineralocorticoid receptor blocker) for hypertension at a local clinic. An abdominal CT scan conducted to investigate secondary hypertension revealed a left adrenal tumor, prompting her to undergo further examination at another hospital. Subsequently, Cushing syndrome was suspected, and she was referred to our hospital for further evaluation and treatment.

## Diagnostic Assessment

On admission, her height was 168 cm, she weighed 71 kg (her maximum weight), and her body mass index was 25.1 kg/m^2^. Despite treatment for hypertension, her blood pressure had increased to 144/90 mmHg. She exhibited characteristic cushingoid features such as a moon face. Laboratory test findings were as follows: eosinopenia (42/μL, ref: 0-490/μL) and elevated total cholesterol (296 mg/dL [7.7 mmol/L], ref: 146-219 mg/dL [3.8-5.7 mmol/L]), with no electrolyte or glucose tolerance abnormalities observed. Her plasma ACTH level was low at 2.5 pg/mL (0.6 pmol/L, ref: 7-63 pg/mL [1.5-13.9 pmol/L]), whereas her serum cortisol level was within the reference range (15.2 μg/dL [419.4 nmol/L], ref: 5.9-17.0 μg/dL [162.8-469.0 nmol/L]). Her serum dehydroepiandrosterone-sulfate (DHEA-S) level was slightly decreased (159 ng/mL [5.5 μmol/L], ref: 180-3910 ng/mL [6.2-135.6 μmol/L]), 24-hour urinary free cortisol was markedly elevated at 302 μg/day (833.2 nmol/day, ref: 11.2-80.3 μg/day [30.9-221.6 nmol/day]), and 24-hour urinary 17-ketosteroid level was normal (7.87 mg/day [ref: 0.51-9.68 mg/day]). Her serum cortisol level during night-time sleep was elevated (16.9 μg/dL [466.3 nmol/L]). An incomplete suppression of serum cortisol level with a 1-mg overnight dexamethasone suppression test (14.4 μg/dL [397.3 nmol/L]) indicated autonomous cortisol secretion. Based on these results, a diagnosis of ACTH-independent Cushing syndrome was made. Plasma renin activity was not low (1.4 µg/L/h [ref: 0.2-2.3 µg/L/h]) and the plasma aldosterone concentration was not high (41.4 pg/mL [1.48 pmol/L], ref: 3.0-82.1 pg/mL [0.11-2.96 pmol/L]), indicating no autonomous aldosterone co-secretion.

**Figure 1.**
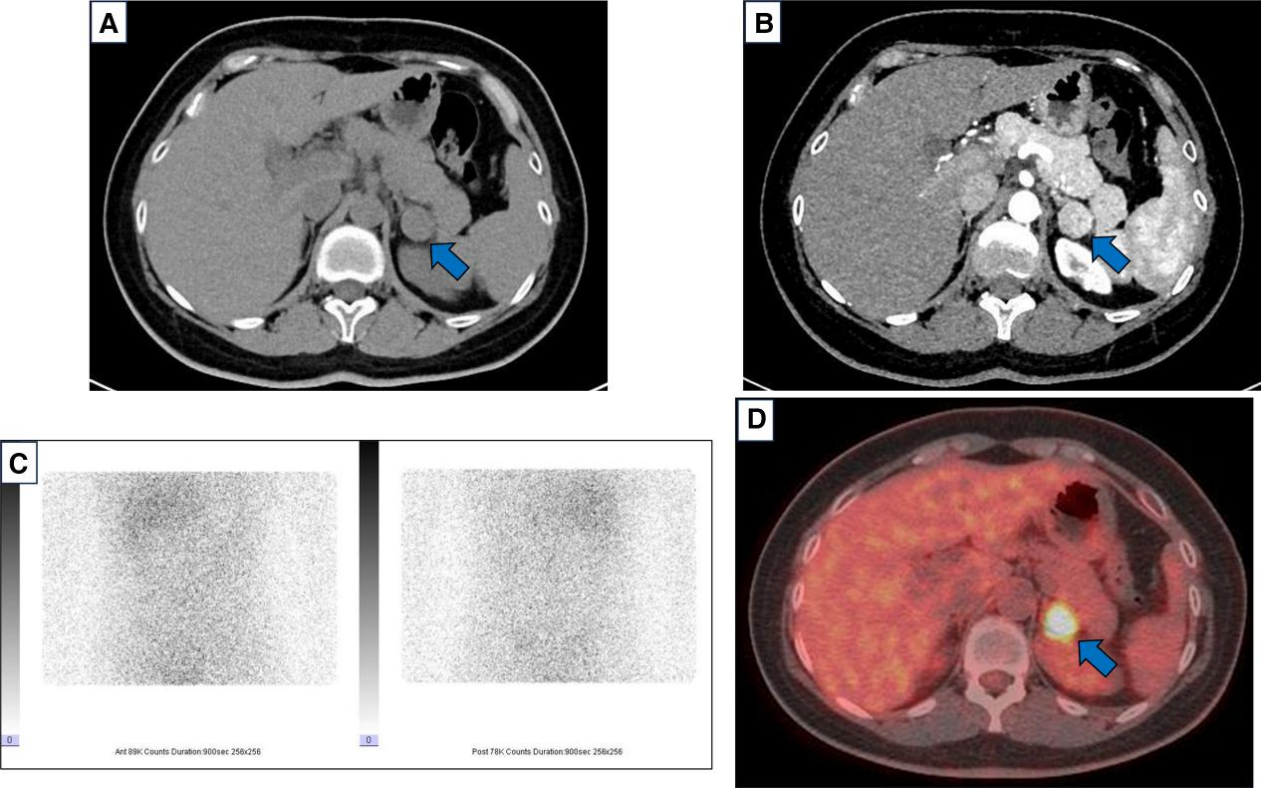
Radiological imaging of the left adrenal tumor. (A) Unenhanced computed tomography (CT) image showing a left adrenal tumor approximately 2 cm in diameter with relatively high density (mean Hounsfield units, 44), (B) Contrast-enhanced CT image showing heterogeneous contrast enhancement of the tumor, (C) ^131^I-adosterol scintigraphy image showing no enhanced isotope accumulation in the tumor, and (D) ^18^F-fluorodeoxyglucose positron emission tomography/CT image showing strong accumulation with maximum standardized uptake value 14.9 at the tumor.

An abdominal CT scan revealed a left adrenal tumor approximately 2 cm in diameter with relatively high density (mean Hounsfield units, 44) (Fig. 1A) and heterogeneous contrast enhancement (Fig. 1B). ^131^I-adosterol scintigraphy showed no enhanced isotope accumulation in the tumor (Fig. 1C), whereas ^18^F-FDG PET/CT showed strong accumulation, with a maximum standardized uptake value (SUVmax) of 14.9 in the tumor (Fig. 1D). These radiological findings are not typical of cortisol-producing adenomas, and we could not rule out the possibility of adrenal carcinoma. Adrenal venous sampling (AVS) was performed to confirm cortisol overproduction in the tumor. Cortisol levels were measured in the adrenal veins (AV) and inferior vena cava (IVC). The cortisol gradient from the AV to the IVC was 0.95 on the right side and 6.55 on the left side. The left AV to right AV gradient of cortisol was 6.89 (serum cortisol level in right AV, 13.2 μg/dL [364.2 nmol/L]; left AV, 91.0 μg/dL [2510.7 nmol/L]; IVC, 13.9 μg/dL [383.5 nmol/L]). As previously reported [8], these results indicated that the left adrenal tumor was the source of excessive cortisol production.

## Treatment

A laparoscopic left adrenalectomy was performed, and the tumor was completely resected.

## Outcome and Follow-up

**Figure 2.**
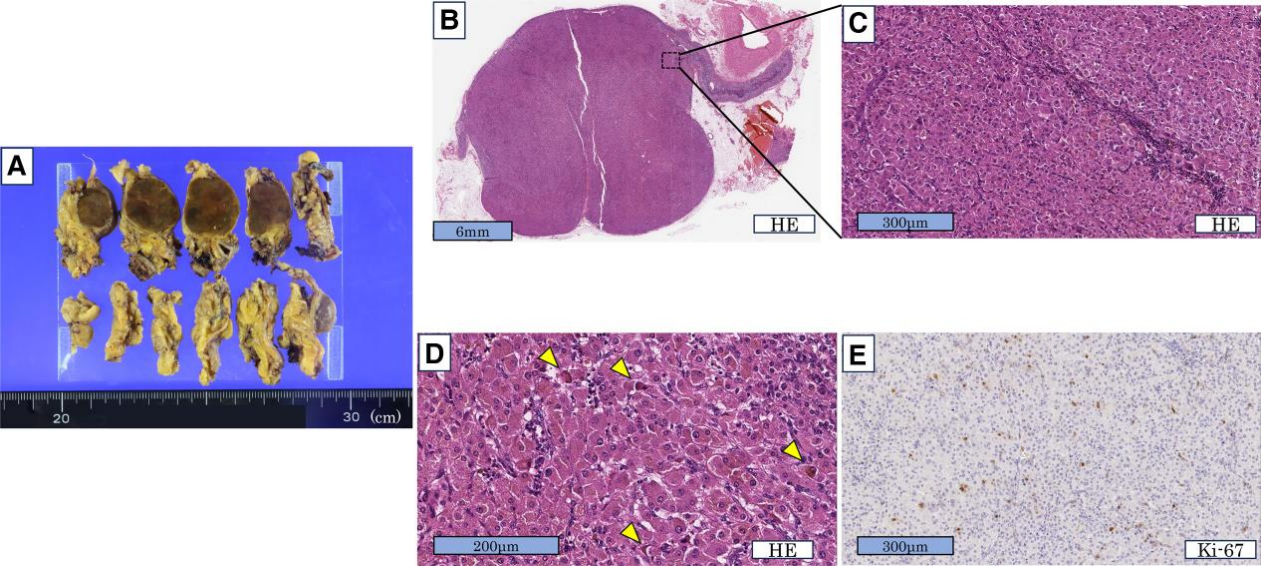
Cut surfaces and light microscopic findings of the resected tumor. (A) Cut surfaces of the resected tumor showing a clearly demarcated, dark brown mass measuring 2 cm in diameter. (B, C) Hematoxylin and eosin (HE) stain of the tumor region at low magnification and high magnification, respectively, showing the tumor predominantly composed of compact cells. (D) HE stain showing some tumor cells with lipofuscin granules (arrowheads). (E) Ki-67 stain indicating the Ki-67 index of 4%.

**Figure 3.**
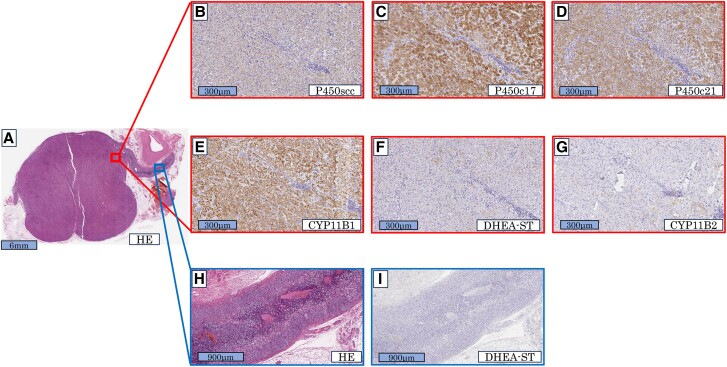
Immunohistochemical analysis of the tumor and adjacent nonneoplastic lesion. (A) Hematoxylin and eosin (HE) stain of the tumor region at low magnification, same as Fig. 2B. (B-G) Immunohistochemical stain showing positive results for P450scc (B), P450c17 (C), P450c21 (D), and CYP11B1 (E), and faintly positive for dehydroepiandrosterone-sulfotransferase (DHEA-ST) (F), and a negative result for CYP11B2 (G). (H, I) HE stain of non-neoplastic adrenal tissue adjacent to the tumor, displaying cortical atrophy and a negative result for DHEA-ST.

Macroscopic evaluation revealed a clearly demarcated, dark brown mass measuring 2 cm in diameter (Fig. 2A). Microscopic evaluation revealed that the tumor was mainly composed of compact cells with abundant eosinophilic cytoplasm and poor lipid content, including scattered lipofuscin granules (Figs. 2B-D). The Ki-67 index was 4% (Fig. 2E), which was compatible with a cortisol-producing adrenocortical adenoma. The lesion was diagnosed as an adrenocortical adenoma according to the Weiss score (1 of 9 points) [9]. Immunohistochemical analysis showed that the tumor cells were positive for P450scc, P450c17, P450c21, and CYP11B1; faintly positive for DHEA sulfotransferase (DHEA-ST); and negative for CYP11B2 (Figs. 3A-G); confirming the cortisol-producing ability of the tumor. The adjacent non-neoplastic adrenal cortex displayed cortical atrophy and was negative for DHEA-ST (Fig. 3H and I), reflecting long-term suppression of the hypothalamus-pituitary-adrenal axis.

Hydrocortisone replacement therapy was initiated postoperatively and gradually tapered. Her blood pressure levels improved postoperatively, leading to antihypertensive drug discontinuation.

## Discussion

Here, we present the case of a young woman with ACTH-independent Cushing syndrome. Her left adrenal tumor exhibited imaging findings atypical for an adenoma, including high unenhanced CT attenuation values, negative uptake on ^131^I-adosterol scintigraphy, and strong accumulation on ^18^F-FDG PET/CT, complicating the preoperative diagnosis. Histopathological analyses indicated a rare type of cortisol-producing adrenocortical adenoma predominantly composed of compact cells. These atypical imaging findings are likely associated with a compact cell-dominant histology.

The first characteristic finding was high unenhanced CT attenuation values. Adrenal adenomas comprise a mixture of lipid-rich clear cells and lipid-poor compact cells in various proportions, and the clear cell/compact cell ratio is inversely correlated with the unenhanced CT attenuation values [10]. Typically, cortisol-producing adenomas show low unenhanced CT attenuation values because of a high proportion of lipid-rich clear cells. However, in our patient, the high proportion of lipid-poor compact cells contributed to the high unenhanced CT attenuation values.

The second notable finding was the negative scintigraphy findings. ^131^I-adosterol scintigraphy has a very high sensitivity and specificity for ACTH-independent Cushing syndrome [11, 12] and is a useful tool for functional evaluation. Radioisotope accumulation appears to depend on the intracellular cholesterol ester pool. Radiopharmaceuticals used in ^131^I-adosterol scintigraphy, ^131^I-6β-iodomethylnorcholesterol, are taken up by adrenocortical cells through low-density lipoprotein receptors and accumulated as cholesterol esters after esterification [13]. Hormone-sensitive lipase (HSL), which catalyzes the conversion of free cholesterol from cholesterol esters, and acyl-CoA: cholesterol acyltransferase 1/2 (ACAT1/2), which catalyzes the conversion of free cholesterol into cholesterol esters, play important roles in maintaining intracellular cholesterol levels [14]. A recent study demonstrated that both HSL and ACAT1/2 activities are low in clear cells, maintaining the intracellular cholesterol ester pool [15]. In contrast, increased HSL activity compared to that of ACAT1/2 in compact cells promotes the conversion of cholesterol esters to free cholesterol, resulting in a shrinking of the intracellular cholesterol ester pool [15]. These findings suggest that the predominance of compact cells in the patient's adenoma resulted in less intracellular accumulation of esterified isotopes, contributing to the negative scintigraphy results.

The third finding was high FDG accumulation. The average SUVmax of cortisol-producing adrenal adenomas in ^18^F-FDG PET/CT is 5.9 ± 3.0, which is higher than that of aldosterone-producing adenomas and nonfunctioning adenomas [16]. However, our patient had a higher SUVmax of 14.9. Cortisol-producing adenomas with a higher SUVmax have been reported to have more compact cells and higher expression of the glucose transporter (GLUT)-1,3, and genes related to glycolysis and aerobic metabolism. In addition, SUVmax and expression of these genes have shown strong positive correlations [17]. Therefore, we consider that the increased FDG uptake via GLUT-1,3 in compact cells led to the high SUVmax in our patient's adenoma.

Similar atypical imaging findings have also been reported in adrenocortical carcinoma and black adenoma [6, 18]. In our patient, ^18^F-FDG PET/CT showed strong accumulation in the left adrenal tumor; however, adrenocortical carcinoma was excluded based on pathological findings. Black adenomas are characterized by abundant compact cells and fewer clear cells with pigmented granules containing lipofuscin in their cytoplasm [19]. Excessive lipofuscin deposition results in a black appearance [19]. Although our patient's adenoma had the same imaging findings as in black adenoma [17], the adenoma did not show a black appearance because of scattered lipofuscin granules. However, it had characteristics similar to those of black adenomas with respect to compact cell predominance. Therefore, we consider that a high proportion of compact cells, regardless of lipofuscin content, can lead to atypical imaging findings, as observed in our patient.

Serum DHEA-S level was marginally decreased but not completely suppressed, despite strong suppression of ACTH. This may also be related to the compact cell predominance. Compact cells are hormonally active and have relatively higher expression of DHEA-ST than clear cells [20]. In our patient, the adjacent non-neoplastic adrenal cortex was immunonegative for DHEA-ST, but the tumor cells were faintly immunopositive for DHEA-ST (Fig. 3F and 3I), which may explain the incomplete suppression of the serum DHEA-S level.

We did not evaluate the tumor using magnetic resonance imaging (MRI), which is a limitation in our case report. Changes in MRI signal intensity on chemical shift images are useful in the diagnosis of adrenal adenomas because they reflect lipid content [2]. However, in adenomas predominantly composed of compact cells, as in our patient, the benefit of MRI may be limited because of the poor lipid content. AVS is usually not required for the diagnosis of ACTH-independent Cushing syndrome in patients with a unilateral adrenal tumor. However, ^131^I-adosterol scintigraphy, which evaluates the laterality of cortisol overproduction, was negative in our patient. There have been a few cases reporting discordant results between CT findings and AVS lateralization, even in patients with a unilateral lesion [21]. Therefore, we performed AVS to confirm cortisol overproduction in the tumor.

In conclusion, we encountered a case of ACTH-independent Cushing syndrome with atypical imaging findings. It is important to recognize in the differential diagnosis that cortisol-producing adrenal adenomas are rarely predominantly composed of compact cells, and such adenomas can present with atypical imaging findings, such as high unenhanced CT attenuation values, negative uptake on ^131^I-adosterol scintigraphy, and strong accumulation on ^18^F-FDG PET/CT.

## Learning Points

- Cortisol-producing adrenal adenomas are rarely predominantly composed of lipid-poor compact cells, displaying atypical radiological features.
- These adenomas can show high unenhanced CT attenuation values, reflecting poor lipid content.
- ^131^I-adosterol scintigraphy can show negative uptake in these adenomas, possibly because of increased cholesterol ester metabolism by HSL.
- A high SUVmax on 18F-FDG PET/CT can be observed, likely from enhanced glucose uptake via GLUT-1,3.

## Data Availability

Original data generated and analyzed for this case report are included in this published article.
